# Carbon accumulation in *Rhodotorula glutinis* induced by nitrogen limitation

**DOI:** 10.1186/s13068-014-0164-0

**Published:** 2014-12-09

**Authors:** Julien Cescut, Luc Fillaudeau, Carole Molina-Jouve, Jean-Louis Uribelarrea

**Affiliations:** Université de Toulouse; INSA,UPS,INP; LISBP, 135 Avenue de Rangueil, F-31077 Toulouse, France; INRA, UMR792, Ingénierie des Systèmes Biologiques et des Procédés, F-31400 Toulouse, France; CNRS, UMR5504, F-31400 Toulouse, France

**Keywords:** Oleaginous yeast, *Rhodotorula glutinis*, Carbohydrate accumulation, Lipid accumulation, C/N ratio, Fed-batch culture

## Abstract

**Background:**

Oleaginous microorganisms, such as bacterium, yeast and algal species, can represent an alternative oil source for biodiesel production. The composition of their accumulated lipid is similar to the lipid of an oleaginous plant with a predominance of unsaturated fatty acid. Moreover this alternative to conventional biodiesel production does not create competition for land use between food and oleo-chemical industry supplies. Despite this promising potential, development of microbial production processes are at an early stage. Nutritional limited conditions, such as nitrogen limitation, with an excess of carbon substrate is commonly used to induce lipid accumulation metabolism. Nitrogen limitation implies modification of the carbon-to-nitrogen ratio in culture medium, which impacts on carbon flow distribution in the metabolic network.

**Results:**

The goal of the present study is to improve our knowledge of carbon flow distribution in oleaginous yeast metabolism by focusing carbon distribution between carbohydrate and lipid pools in order to optimize microbial lipid production. The dynamic effects of limiting nitrogen consumption flux according to carbon flow were studied to trigger lipid accumulation in the oleaginous yeast *Rhodotorula glutinis*. With a decrease of the specific nitrogen consumption rate from 0.052 Nmol.Cmol_X_^−1^.h^−1^ to 0.003 Nmol.Cmol_X_^−1^.h^−1^, a short and transitory intracellular carbohydrate accumulation occurred before the lipid accumulation phase. This phenomenon was studied in fed-batch culture under optimal operating conditions, with a mineral medium and using glucose as carbon source. Two different strategies of decreasing nitrogen flow on carbohydrate accumulation were investigated: an instantaneous decrease and a progressive decrease of nitrogen flow.

**Conclusions:**

Lipid production performance in these fed-batch culture strategies with *R. glutinis* were higher than those reported in the previous literature; the catalytic specific lipid production rate was 0.07 Cmol_lip_.Cmol_X*_^−1^.h^−1^. Experimental results suggested that carbohydrate accumulation was an intrinsic phenomenon connected to the limitation of growth by nitrogen when the nitrogen-to-carbon ratio in the feed flow was lower than 0.045 Nmol.Cmol^−1^. Carbohydrate accumulation corresponded to a 440% increase of carbohydrate content. These results suggest that microbial lipid production can be optimized by culture strategy and that carbohydrate accumulation must be taken account for process design.

## Introduction

The environmental and economic impacts of fossil fuel uses have stimulated research into renewable biofuel production. Rising prices of crude oil, depletion of resources and directives imposing reductions in greenhouse gas emission have led to increasingly attractive alternative renewable fuels. Focusing on road transportation, with ever growing diesel fuel consumption, biodiesel production [1] from plant oils remains more expensive than petroleum-based diesel due to the high costs of raw materials, as reported by the 2008 Organization for Economic Cooperation and Development (OECD) report. Moreover, conventional biodiesel production creates competition for land use between food and oleo-chemical industry supplies. Research on new routes for fatty acid production from renewable resources is a challenge necessary to improve the competitiveness of the European biodiesel industry and our energy independence [2,3].

Taking these issues into account, an alternative way to produce fatty acids is microbial conversion of carbohydrates from lignocellulosic biomass by oleaginous microorganisms. According to recent works [4-7], microbial oil production constitutes a prospective alternative oil feedstock needing further investigations. In all types of microbial cells, non-polar lipids are accumulated as a source of building blocks needed for membrane formation and energy storage. As reviewed by Ratledge [8], oleaginous yeasts are able to accumulate lipids to up to 70% of their dry cell weight. Examples are *Rhodosporidium sp*., *Rhodotorula sp*. and *Lipomyces sp*. More precisely, they mainly synthesize and store triacylglycerols (TAG) and steryl esters (SE) [9,10]. Triacylglycerols (TAG) are composed of fatty acids with aliphatic tails of 16 to 18 carbons, saturated and unsaturated (up to two unsaturations), perfectly compatible with use as fuels [2]. According to previous works [11,12], lipid production requires an excess of carbohydrate substrates (for example glucose, glycerol, polysaccharides and so on) under nutrient depletion (mainly nitrogen, phosphorus, zinc, iron and magnesium) [13,14]. More generally, nutrient depletion has a negative impact on different levels of microorganism activity. Several works have studied the impact of nitrogen starvation on yeast activity and established a strong slowdown of cell multiplication [15,16], fermentation activity and substrate uptake capacity [17]. Moreover, chitin accumulation (C_8_H_13_O_5_N)_n_ was reported during batch cultures of *Rhodotorula glutinis* without accurate nitrogen limitation control [18]. Chitin is a structural polysaccharide of fungal cell walls. Therefore, under nitrogen depletion, carbon fluxes could obviously be distributed between lipid and carbohydrate storages.

In a natural biotope, microorganisms are placed in a physical and chemical environment in which capacity to store carbon is an advantage. Carbon can be stored by microorganisms in two main forms: lipid and carbohydrate. The simultaneous biological phenomena involved in lipid and carbohydrate storage have been the object of very few studies in yeast.

The goal of the present study is to improve our knowledge of carbon flow distribution in oleaginous yeast metabolism by focusing carbon distribution between carbohydrate and lipid pools in order to optimize microbial lipid production as an alternative means of biodiesel production. The basidiomycetous yeast *R. glutinis* CECT 1137 is a relevant microorganism model for studying lipid accumulation, as a final cell concentration of 185 g dry cell weight.L^−1^ with 40% (g of lipid per g of dry cell) in 84 hours was achieved during fed-batch culture aerated with oxygen-enriched air [19].

Different cultures were carried out to quantify the influence of two nitrogen limitation strategies on dynamic biological phenomena, in well-controlled fed-batch culture with glucose as sole substrate. The first strategy consisted in suddenly stopping the nitrogen supply (experiment (a)), and the second was a sigmoidal reduction of the nitrogen supply (experiment (b)).

## Materials and methods

### Microorganism and growth media

*R. glutinis* CECT 1137 wild-type was supplied by the Spanish Type Culture Collection (Coleccion Espanola de cultivo tipo), University of Valencia, Spain.

Axenic cultures were carried out and harvested before the stationary growth phase in shaken flask cultures on lysogeny broth (LB) rich medium containing 10 g.L^−1^ of glucose. After addition of sterile glycerol solution (30% v/v), 1 mL aliquots were stored in sterile vials at –80°C. These frozen stock cultures were used to inoculate precultures for all fed-batch experiments.

Inocula were prepared as follows: batch culture was performed in 100 mL baffled Erlenmeyer flasks containing 8 ml of LB rich medium at 30°C for 16 hours on a rotary shaker (100 rpm). The culture was transferred to a 250 mL baffled Erlenmeyer flask containing 72 mL of mineral medium (pH 5.5). The composition of mineral medium for preculture and culture, defined according to the method of Egli and Fiechter [20] was, in g.L^−1^: KH_2_PO_4_, 4.54; (NH_4_)_2_HPO_4_ 0.83; (NH_4_)_2_SO_4_, 2.47; MgSO_4_ · 7H_2_O, 1.7; ZnSO_4_ · 7H_2_O, 0.016; FeSO_4_ · 7H_2_O, 0.0701 MnSO_4_ · H_2_O, 0.0029; CoCl_2_ · 6H_2_O, 0.025; CuSO_4_ · 5H_2_O, 0.0031; Na_2_MoSO_4_ · 2H_2_O, 0.0012; CaCl_2_ · 2H_2_O, 0.018; NaCl, 0.040 and 1 ‰ (v/v) vitamin solution. The composition of the vitamin solution was, in g.L^−1^: d-biotin, 0.05; thiamine hydrochloride, 1; pantothenic acid, 1; pyridoxol hydrochloride, 1; nicotinic acid, 1; p-aminobenzoic acid, 0.2 and myo-inositol, 25. The pH of the medium was adjusted to the working pH (5.6) with phosphoric acid and the initial glucose concentration was 10 g L^−1^. After 12 hours of growth at 30°C, the 80 ml culture broth was used to inoculate a 5 L Erlenmeyer flask containing 720 mL of mineral medium with vitamins as described above and incubated at 30°C for 12 hours. The latter culture was used to inoculate 7.2 L of the mineral medium in the bioreactor.

The bioreactor was supplied with a constant flow rate ratio of mineral medium feed to substrate feed of 1:10. The composition of the feeding mineral medium was, in g.L^−1^: KCl, 7.45; CuSO_4_ · 5H_2_O, 0.009; Na_2_MoO_4_ · 2H_2_O, 0.0360; MgSO_4_ · 7H_2_O, 5.278; CaCl_2_ · 2H_2_O, 0.051; ZnSO_4_ · 7H_2_O, 0.078; FeSO_4_ · 7H_2_O, 0.215; MnSO_4_ · H_2_O, 0.009; H_3_BO_3_, 0.0025; CoCl_2_ · 6H_2_O, 0.158; H_3_PO_4_, 28.62 and H_2_SO_4_, 16.370. Vitamin solution was added correlated to biomass growth according to Alfenore *et al*. [21]; 10 mL of vitamin mixture were added when 10 g_X_. L^−1^ of biomass was formed.

Vitamins were all purchased from Sigma-Aldrich Chimie (Lyon, France) with a purity of at least 99.9%. Glucose was provided by Roquette (France, Lestrem). All other chemicals (mineral medium, solvent, mobile phases and so on) were purchased from VWR (Fontenay sous bois, France) with a purity of at least 99%.

### Culture

Fed-batch cultures were performed in a 20 L bioreactor using the Braun Biostat E fermenting system (Braun, Melsungen, Germany) without oxygen limitation. The temperature was held at 30°C, and the pH at 5.6, with the addition of a 10 mol.L^−1^ NH_3_ solution (growth phase) or KOH solution (lipid-accumulation phase). Homemade software enabled the online acquisition and regulation of the controlled parameters of the bioreactor (stirring speed, pH, temperature, relative pressure, partial pressure of dissolved oxygen (DO), bases and antifoam additions). The relative pressure in the bioreactor was set at 0.3 bar. The maximum amount of antifoam (Struktol JG73) added during culture was 0.5 ml. The bioreactor was supplied with three sterile feeds (glucose, salt and base (ammonium or potassium hydroxide)) using peristaltic pumps (Masterflex (France, Illkirch) and Gilson (United States, WI, Middleton). The glucose feeding concentration was 730 g.L^−1^. The masses of glucose and nitrogen solutions added into the fermentor were estimated online by weighing the feeding tanks continuously (CPA16001S, Sartorius (Goettingen, Germany). Outlet gas was analyzed by mass spectrometry after the gas condenser. The mass spectrometer (Prima 600 s, VG Gas, Manchester, United Kingdom) was used for its accuracy to measure CO_2_, O_2_, N_2_ and Ar compositions. The gas flow was ionized by electron bombardment, and the components separated according to their mass-to-charge ratio by applying a magnetic field, then collected by a detector. O_2_ consumption rate and CO_2_ production rate were calculated from mass balances, taking into account the variation of the gas volume in the reactor, inlet airflow (measured by a mass flowmeter, Brooks, United States,PA, Hatfield), temperature, humidity and pressure. The glucose concentration in the fermentor was evaluated online by homemade software based on carbon mass balance, taking into account online acquisition data.

### Culture strategy

Fed-batch cultures were performed according to three phases with different carbon and nitrogen feeding strategies: (I) growth phase without nitrogen limitation, (II) transient phase of depletion of residual nitrogen and (III) growth and production phase under nitrogen limitation.

During phase (I), an exponential glucose flow was imposed to give a constant specific growth rate defined as the variation of cell mass per unit time per cell mass. Nitrogen requirements were ensured by pH regulation to keep the pH constant at 5.6 with NH_4_OH solution.

**Figure 1:**
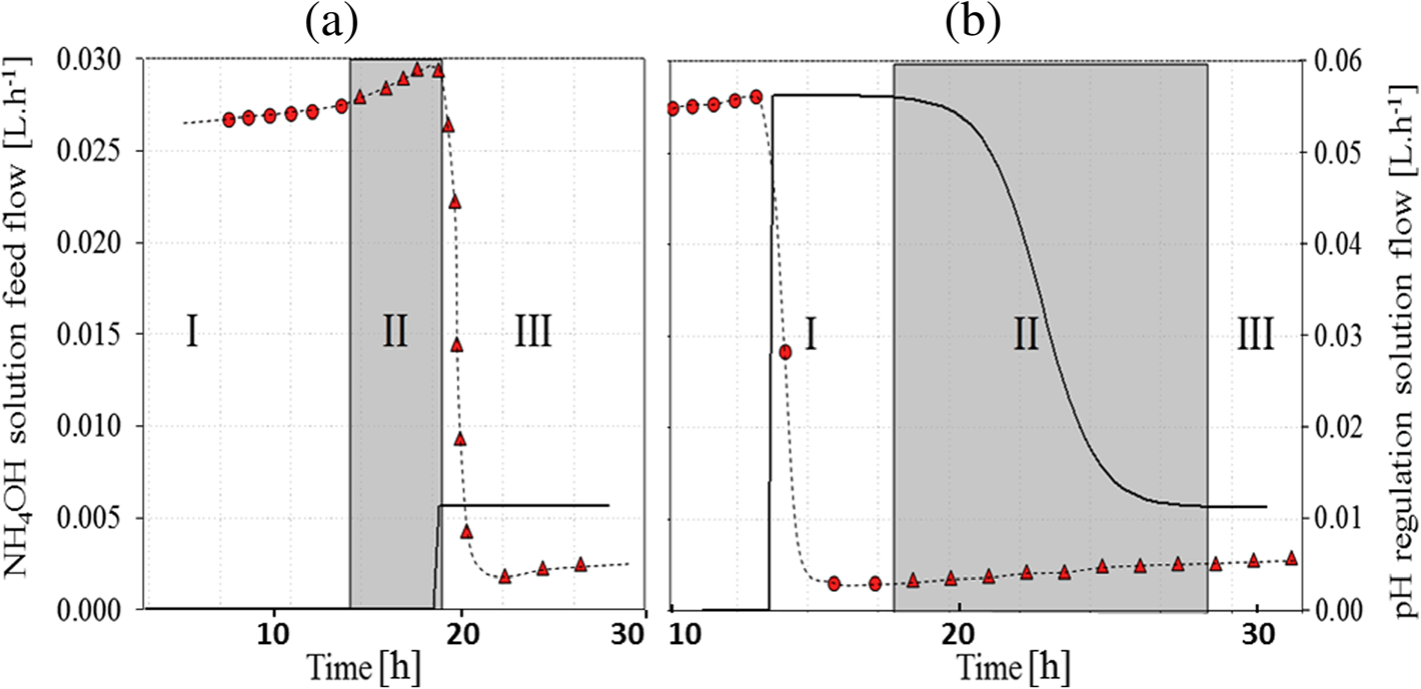
**Evolution of NH**_**4**_ **OH solution feed flow (L.h** ^**−1**^ **; solid line) versus time and pH regulation solution flow (symbol and dotted line) versus time.** Circle represents NH_4_OH solution, triangle represents KOH solution. **(a)** culture with instantaneous stop of nitrogen flow, **(b)** sigmoid decrease of nitrogen flow.

Phase (II) corresponded to nitrogen limitation with an excess of carbon source. It started by swapping NH_4_OH pH regulation to potassium hydroxide solution (10 mol.L^−1^). This shift simultaneously held the pH at the setpoint and decreased nitrogen concentration in the broth. Two strategies were carried out in order to set up nitrogen limitation: culture (a): a sudden stop of the nitrogen supply was applied by swapping the pH regulation source and culture (b): a sigmoid reduction of the nitrogen supply was performed while changing pH regulator, and the NH_4_OH solution feed flow Q_N_(t) was controlled according to the sigmoid curve in Figure 1.

1 $$ {Q}_n(t)-Q{}_n\left({t}_0\right)+{Q}_n\left({t}_f\right)\times \frac{1}{\begin{array}{l}-\frac{t-{t}_0-\frac{\varDelta t}{2}}{\frac{\varDelta t}{10}}\\ {}1+e\end{array}} $$

Equation 1 Equation of NH4OH solution feed flow $\mathbf{Q}\mathbf{N}\left(\mathbf{t}\right)\kern0.24em \left[\boldsymbol{m}{\boldsymbol{L}}_{\boldsymbol{N}{\boldsymbol{H}}_4\boldsymbol{O}\boldsymbol{H}}.{\mathbf{\mathsf{h}}}^{\mathbf{\hbox{-}}\mathbf{\mathsf{1}}}\right]$ at time t [h] with t_o_: time of the beginning of phase II of culture (b); [h], t_f_: time of the end of phase II of culture (b); [h], Δt: duration of phase II; [h].

For both culture types (a) and (b), the phase III was characterized by a constant nitrogen limitation resulting in exponential nitrogen and substrate feed. Phase III started when nitrogen concentration in the broth was below 10 mmol.L^−1^ and NH_4_OH flow controlled the value of the specific growth rate of 0.05 h^−1^. This value allowed optimal lipid synthesis and yeast viability as reported previously by Su *et al*. [22]. The glucose flow was adjusted to allow both residual growth (according to ammonium feeding flow rate) and lipid neo-synthesis demands.

**Figure 2:**
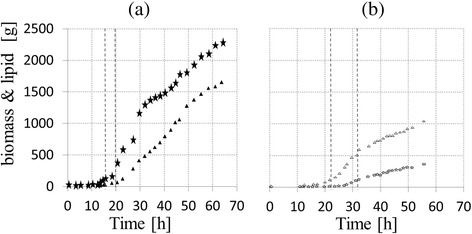
**Evolution versus time of cell (stars) and lipid (triangles) masses [g] for (a) culture with instantaneous stop of nitrogen flow (b) culture with sigmoid decrease of nitrogen flow.**

An example of profiles of nitrogen feeding strategies and flows of alkali control pH are reported in Figure 2.

### Analytical methods

Yeast concentration was evaluated by spectrophotometric measurements at 600 nm in a Hitachi U-1100 spectrophotometer (Japan, Tokyo, Chiyoda-ku) with a 0.2 cm optic path cell. For each sample, the average of three measurements was calculated.

For cell dry weight determination, culture samples (1 to 10 ml) were harvested by filtration on a 0.45 μm membrane (Sartorius (Deutschland, Goettingen) and dried at 200 mm Hg and 60°C for 48 hours until constant weight.

The unit of the dry cell weight was noted g_dcw_ and the unit of cell concentration : g_dcw_.L^-1^. Ash was determined following two complete combustions with 200 μL of NH_4_NO_3_ solution (20 g.L^−1^) in a muffle furnace at 550°C for 12 hours each.

The biomass formula was determined by elemental analysis of C, H, O and N. CH_1.650_O_0.497_ N_0.135_ was taken for yeast composition during growth phase. Based on the lipid composition profile, an average lipid formula of CH_1.820_O_0.105_ was calculated and used for mass balances and rates calculations. Corresponding molar mass was 15.53 g.Cmol^−1^.

Sugar concentrations from supernatants were determined by HPLC (Ultimate 3000, Dionex (United States, CA, Sunnyvale) using an Aminex HPX-87H + column (300 × 7.8 mm) with the following conditions: a temperature of 50°C, with 5 mM H_2_SO_4_ as eluant (flow rate of 0.5 ml min^−1^) and dual detection, refractometer (Shodex (United States, NY, New York) and UV at 210 nm (Dionex (United States, CA, Sunnyvale). Compounds were identified and quantified with reference to standards. Glucose concentration from culture supernatants was also determined during culture with a glucose analyser YSI model 27 A (Yellow Springs, United States, OH, Yellow Springs).

To quantify the residual nitrogen concentration in the culture medium, an ammonium ion electrode (PH/ISE meter model 710A + Ammonia Gas Sensing Electrode Model 95-12, Orion Research Inc, Boston, United States) was used.

Extraction of total cellular lipids from lyophilized sample was performed using the PLE system, ASE 300 (provided by Dionex) as described by Cescut *et al*. [23]. Briefly, three cycles of 15 minutes at 100°C with a ratio of 144 g of Hydromatrix (Varian (United States, CA, Palo Alto)) per 100 g of dry cell weight three. Extraction cycles used three different chloroform-to-methanol solvent mixtures: 1:2; 1:1 and 2:1 (v/v). For each solvent mixture, two static cycles were applied. To eliminate non-lipid components, the organic phase was shaken against a KCl solution (0.08 g.L^−1^) with a ratio of 25% (v/v) for 15 minutes, then a liquid-to-liquid separation technique was used after centrifugation (5,000 g, 10 minutes). Finally, lipids were recovered as dry material after solvent evaporation in a rotavapor (35°C). Total lipid content was determined by weighing. The lipid content was calculated as the ratio of lipid mass on total dry cell weight.

Total carbohydrate was determined chemically by an adaptation of the phenol-sulfuric acid method as described by Dubois *et al*. [24]. Cells were washed three times with physiological saline at 0°C to eliminate carbohydrate from the supernatant. Yeast suspension (1 mL) was mixed with 1 mL phenol solution (5% w/v) followed by addition of 5 mL concentrated sulphuric acid. The sample was shaken for 30 seconds and maintained at 30°C for 20 minutes prior to measuring absorbance at 486 nm using a spectrophotometer (spectrophotometer Hitachi U-1100). The total mass of carbohydrate was determined based on a standard calibration curve prepared using glucose and mannose. Since the colorimetric response was dependent on the nature of the sugar, a mannose-to-glucose ratio of 1:1 was used as standard, like the average ratio between β-glucans and mannans in the yeast cell walls as mentioned by Klis *et al*. [25]. All analyses using the phenol-sulfuric acid method were performed in quadruplicate.

With the progress of the reaction, due to nitrogen limitation and high intracellular accumulation of carbon, ash content and elementary composition of biomass varied considerably during different phases of culture. To quantify conversion yield of substrate into different products such as lipid and carbohydrate, the unit Cmole was useful, as pointed out by Roca *et al*. [26]. The amount of substance of total biomass (X), expressed in Cmol_X_, was considered as the sum of the amount of substance of elementary catalytic biomass (X^*^) determined in Phase I and the amount of substance in accumulated pools of lipids (lip_acc_) and carbohydrates (carbo_acc_). Catalytic biomass was considered as biomass without accumulated lipid and accumulated carbohydrate. Specific production of compound rates (defined as the variation of compound mass per unit time per cell mass) were calculated considering catalytic biomass concentration and named catalytic specific production rate (q*).

For all experiments, carbon and redox balances were recovered with a deficit of less than 5%.

## Results and discussion

**Figure 3:**
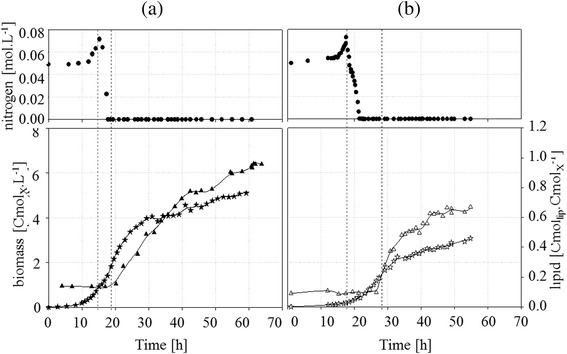
**Variation during fed-batch culture of** ***R. glutinis*** **at 30°C with (a) sudden nitrogen limitation and (b) progressive limitation of nitrogen (circles; [Nmol.L** ^**−1**^ **]) and biomass concentrations (stars; [Cmol**_**X**_ **.L** ^**−1**^ **]) and intracellular lipid amounts (triangles; [Cmol**_**lip**_ **.Cmol**_**X**_^**−1**^ **]). Vertical dotted lines symbolize the beginning of phases II and III.**

As mentioned above, the aim of this work was to study the influence of nitrogen limitation on intracellular carbon storage in the oleaginous yeast *R. glutinis*. In order to characterize yeast physiology, carbon and nitrogen consumption, growth and distribution of carbon among macromolecular pools were quantified during fed-batch culture under perfectly controlled conditions. All fed-batch cultures were performed in duplicate. The evolution versus time of cell and lipid mass for both cultures are shown in Figure 3. For culture (a) 2,300 g_dcw_ of yeast were produced with 1,600 g of lipids in 63 hours. For culture (b), the growth rate was reduced to precisely analyze the nitrogen limitation onset. This is why biomass and lipid productions were lower: 1,100 g_dcw_ of yeast and 400 g of lipids in 55 hours. The evolutions of nitrogen and biomass concentrations and the amount of intracellular lipid versus time during fed-batch culture for both strategies are shown in Figure 3.

### The catalytic biomass: phase I

Macromolecular and elementary composition of catalytic biomass with a balanced metabolism (only growth, no lipid accumulation) was characterised during phase I. Due to the limiting glucose flow, growth was exponential for both cultures.

With the appropriate exponential feed of carbon source, the specific growth rate (μ) was nearly the same and maintained at 0.28 h^−1^ ± 0.01 for culture (a) and at 0.27 h^−1^ ± 0.01 for culture (b); the residual glucose concentrations were lower than 0.01 ± 0.004 Cmol.L^−1^ and 0.10 ± 0.004 Cmol.L^−1^ respectively. For both experiments, ammonium ion accumulated slowly in the broth linked to the neutralization of acid from the salts in the feed solution; the nitrogen concentration increased from 50 to 70 mmol L^−1^ during phase I (Figure 3).

For both cultures, the ratio between consumed carbon flow and consumed nitrogen flow (the C:N ratio) was 0.11 ± 0.02 mol.Cmol^−1^ during phase I; catalytic specific glucose consumption rate was 0.53 ± 0.05 Cmol_glc_.Cmol_X*_^−1^.h^−1^. Specific nitrogen consumption rate was stable around 0.052 mol_N_.mol_X*_^−1^.h^−1^ ± 0.005 mol_N_.mol_X*_^−1^.h^−1^ for culture (a) and (b).

For both cultures, in phase I, the exponential phase of growth, stability of biomass yields and kinetic parameters suggested that the physiological behaviour and composition of macromolecules in cells were similar. Until nitrogen limitation occurred, the composition of the catalytic biomass was CH_1.84_O_0.60_ N_0.17_.

**Figure 4:**
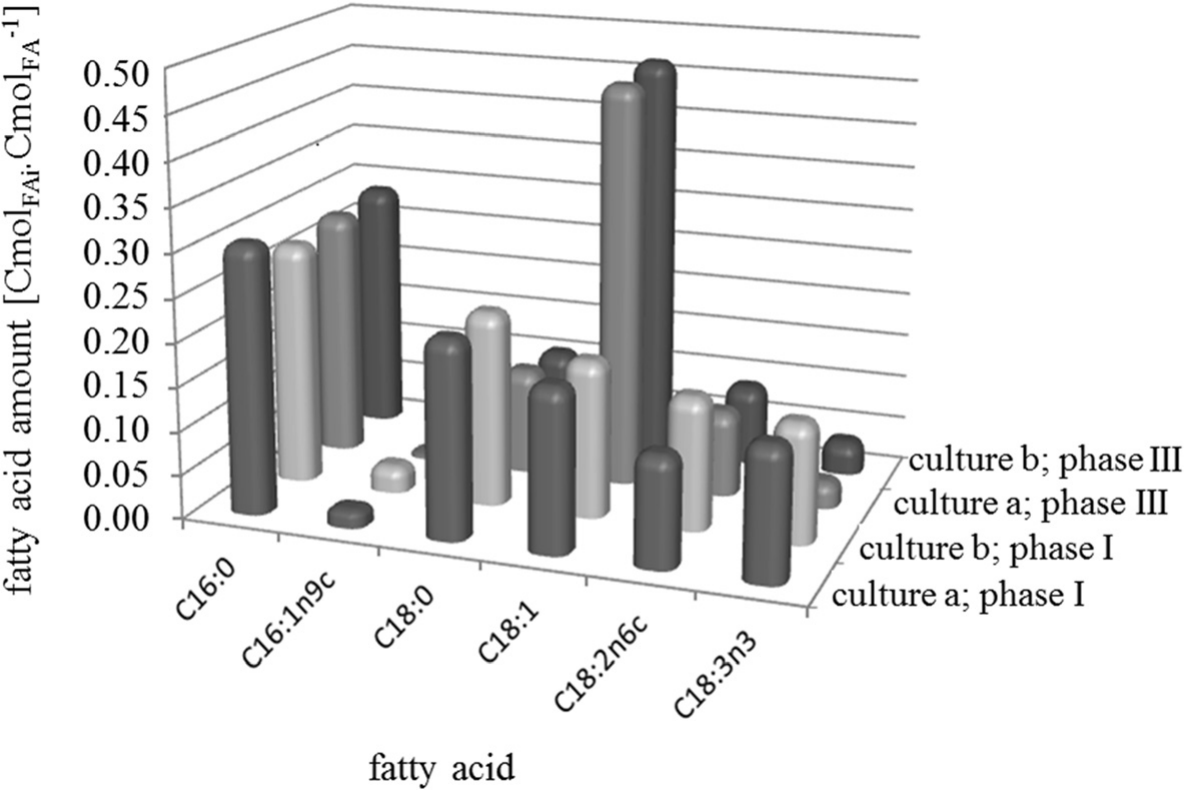
**Average fatty acid profiles of intracellular lipids during culture (a) and (b).** In the front of the panel: average fatty acid profiles during exponential growth (phase I) of fed-batch culture of *R. glutinis* at 30°C with sudden nitrogen limitation (culture **(a)**) and progressive limitation (culture **(b)**) . In the rear of the panel: average fatty acid profiles during nitrogen limitation (phase III) of fed-batch culture of *R. glutinis* at 30°C with sudden nitrogen limitation (culture **(a)**) and progressive limitation (culture **(b)**).

Intracellular lipid content in catalytic biomass, for both culture (a) and (b), was constant and equal to 0.11 Cmol_lip_.Cmol_X*_^−1^ ± 0.02. This usual value is consistent with Ratledge *et al*. [27]. Figure 4 shows the fatty acid composition of intracellular lipids in both cultures during phase I. Fatty acid profiles were similar for both cultures with a majority of palmitic acid (C16:0, 0.31 Cmol_C16:0_.Cmol_FA_^−1^) followed by stearic acid (C18:0, 0.23 Cmol_C18:0_.Cmol_FA_^−1^) which gives a degree of lipid unsaturation of 0.90 for culture (a) and 0.92 for culture (b).

**Figure 5:**
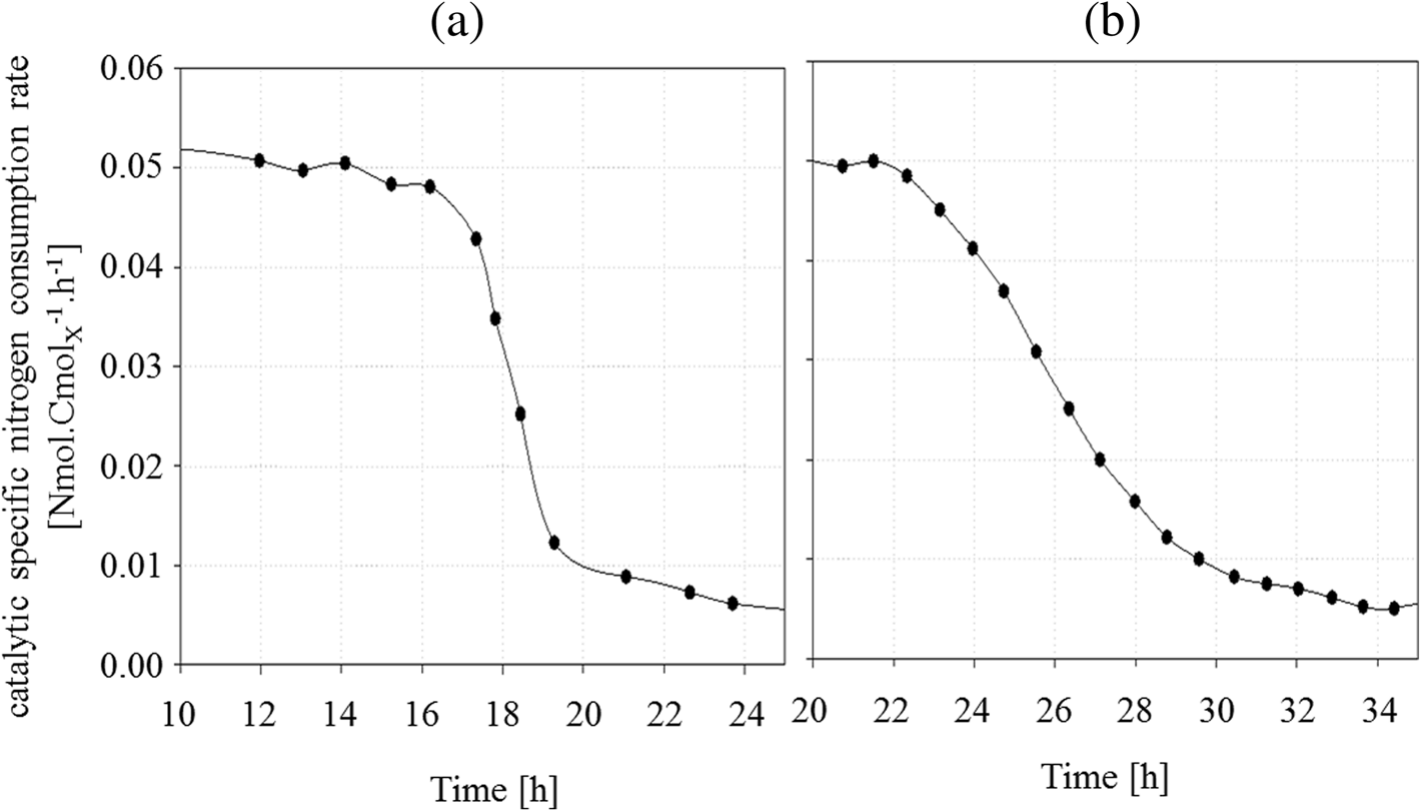
**Evolution of Carbohydrate content for culture (a) and (b).** On left : evolution of carbohydrate content (Cmol_glc_ Cmol_X_^−1^) versus time with sudden nitrogen limitation (**a**, solid dots) on right: evolution of carbohydrate content versus time with a progressive limitation (**b**, empty dots) during fed-batch culture of *R. glutinis* at 30°C.

Accumulated carbohydrate content for culture (a) and (b) is plotted versus time in Figure 5 in Cmol equivalent glucose ([Cmol_glc_.Cmol_X_^−1^]). During phase I, the amount of accumulated carbohydrate was stable at around 0.09 Cmol_glc_.Cmol_X_^−1^.

### Dynamic effect of limiting nitrogen consumption flux: phases II and III

#### Substrate consumption: glucose and nitrogen

The duration of phase II was 3.6-fold longer in culture (b), with a progressive nitrogen limitation onset, than in culture (a) (12.5 versus 3.5 hours). During phase II residual glucose concentration was 0.06 ± 0.004 Cmol.L^−1^ in culture (a) and 0.10 ± 0.004 Cmol.L^−1^ in culture (b); during phase III, they were equal to 0.3 ± 0.004 Cmol.L^−1^ for culture (a) and 0.18 ± 0.3 Cmol.L^−1^ for culture (b). These low residual glucose concentrations showed that carbon flow values were suited to yeast carbon demand for growth, carbohydrate and lipid synthesis.

Whatever the nitrogen limitation onset strategy in phase II, the N:C ratio flux decreased from 0.11 mol.Cmol^−1^ (end of phase I) to 0.03 mol.Cmol^−1^ (start of phase III). For both cultures, the end of phase II corresponded to an extracellular nitrogen concentration below 0.01 mol.L^−1^. Just after this, the nitrogen concentration was below 2 mmol L^−1^ (detection threshold of quantification method), whereas the nitrogen feed was continuous.

In terms of catalytic specific rates, catalytic specific glucose consumption rate decreased from 0.53 ± 0.05 Cmol_glc_.Cmol_X*_^−1^.h^−1^ (14 hours, end of phase I) to 0.11 ± 0.005 Cmol_glc_.Cmol_X*_^−1^.h^−1^ (start of phase III). These values were close to those mentioned by Granger *et al*. [28], equal to 0.5 and 0.1 Cmol_glc_.Cmol_X*_^−1^.h^−1^ respectively. This decrease of catalytic specific glucose consumption rates could be explained by nitrogen limitation controlling specific growth rate and feedback regulation at glycolysis level. During lipid accumulation, citrate, as an intermediate metabolite of fatty acid synthesis, has a negative action on phosphofructokinase regulation. This reaction is the primary step for allosteric enzyme regulation of glycolysis [29]. The inhibition induced a decrease of glucose demand [30].

**Figure 6:**
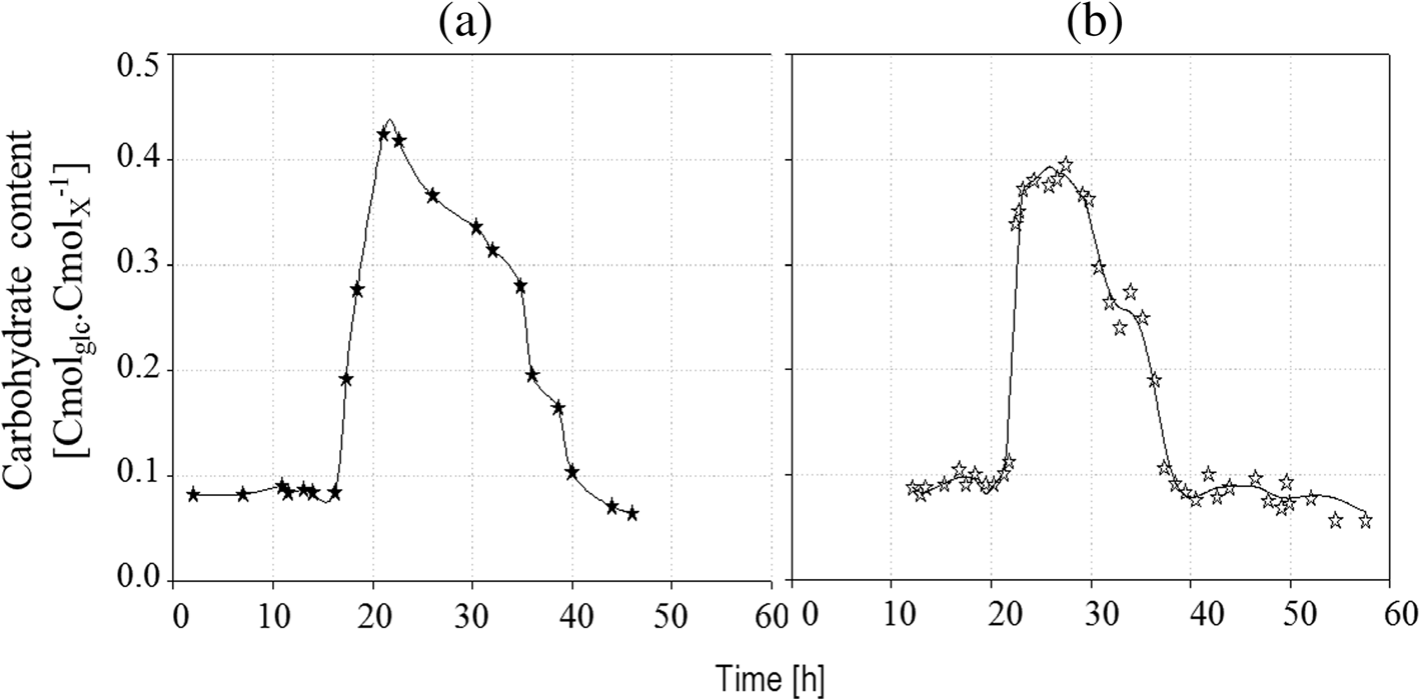
**Evolution of catalytic specific nitrogen consumption rate for culture (a) and (b).** Evolution of catalytic specific nitrogen consumption rate (q*_N_; (Nmol.Cmol_X*_^−1^_._h^−1^)) versus time during fed-batch culture of *R. glutinis* at 30°C with sudden nitrogen limitation **(a)** and progressive limitation **(b)**.

As shown in Figure 6, for culture (a), during the transition phase, specific nitrogen consumption rate was divided by 5 in 3 hours to reach 0.006 Nmol.Cmol_X*_^−1^.h^−1^ at 24 hours. For culture (b), specific nitrogen consumption rate decreased following the sigmoidal nitrogen feed flow, with a delay of 20 minutes. The value 0.006 Nmol.Cmol_X*_^−1^.h^−1^ was reached at 34 hours.

#### Biomass production

During phase II, total biomass concentration was multiplied by 2.5 for culture (a) and by 7 for culture (b): 93.5 Cmol_X_ of total biomass were produced during whole culture (a) and 47 Cmol_X_ during whole culture (b). For culture (a), during phase II, from 15 to 19 hours, due to nitrogen flow decrease, the specific growth rate μ decreased hyperbolically from 0.28 h^−1^ to 0.10 h^−1^. For culture (b), during phase II, between 17.5 and 28 hours, μ decreased sigmoidally from 0.26 h^−1^ to 0.09 h^−1^. For cultures (a) and (b), μ decreased slowly to 0.05 h^−1^ during whole phase III, in agreement with the optimum value for lipid accumulation activity of *R. glutinis* reported by Granger *et al*. [28].

#### Carbon distribution between lipid and carbohydrate pools

Lipid accumulation started around 5 hours (culture (a)) and 8 hours (culture (b)) after the start of nitrogen limitation in phase III. This lipid accumulation finally reached 0.5 Cmol_lip_.Cmol_X*_^−1^ for culture (a) and 0.68 Cmol_lip_.Cmol_X*_^−1^ for culture (b). The mass of triacylglycerol represented 40% of total lipid mass in phase I and increased up to 80% during phase III for both cultures (data not shown), revealing that accumulated lipids were mainly triacylglycerols. As shown in Figure 4, fatty acid profiles were similar in cultures (a) and (b). Lipid compositions were more unsaturated during the lipid accumulation phase than during the exponential growth phase; oleic acid was the most abundant among the fatty acids accumulated.

Depending on the specific lipid production rate of culture (a) q*_lip_, lipid accumulation started suddenly; the highest catalytic specific production rate of the culture was 0.070 Cmol_lip_.Cmol_X*_^−1^.h^−1^ for culture (a) and 0.065 Cmol_lip_.Cmol_X*_^−1^.h^−1^ for culture (b). With a similar initial nitrogen limitation (0.04 mol.Cmol^−1^), Granger *et al*. [28] reported a specific lipid production rate of 0.06 Cmol_lip_.Cmol_X_.^−1^.h^−1^. During phase III, q*_lip_ decreased to 0.02 Cmol_lip_.Cmol_X*_^−1^ h^−1^ for culture (a) and to 0.021 Cmol_lip_.Cmol_X*_^−1^ h^−1^ for culture (b), whereas substrate to lipid conversion yield remained stable at around 0.42 Cmol_lip_.Cmol_glc_^−1^ for both cultures. The specific lipid production rate decrease could be linked to the nitrogen depletion or to an active regulation control system. Firstly, protein turnover needs a constant nitrogen supply. Under nitrogen limitation, autophagy could take place [31]. Autophagy consists of the degradation of cytoplasmic components including a significant amount of proteins and rRNA. The resulting reduction of catalytic power implies an increase of maintenance value and a decrease of conversion yields. On the other hand, lipid accumulation leads to upheavals in macromolecular composition which impacts intracellular physical and chemical properties: pH, osmotic pressure, steric constraints. Physical accumulation limits could be reached and a superior regulation system may control lipid accumulation metabolism. Considering the whole culture, volumetric productivity was 1.05 g_lip_.L^−1^.h^−1^ for culture (a) and 0.95 g_lip_.L^−1^.h^−1^ for culture (b).

As shown in Figure 5, transitory intracellular carbohydrate accumulation was observed without any accumulation in the broth between 16 and 41 hours. During all cultures, carbohydrate amount increased from 0.09 to 0.40 Cmol_glc_ Cmol_X_^-1^ in culture (a) from 16 to 21 hours, and from 0.1 to 0.38 Cmol_glc_ Cmol_X_^−1^ in culture (b) from 21 to 23 hours.

Then, regardless of the dynamics of the nitrogen limitation set up, carbohydrate accumulation was linked to nitrogen limitation degree or relative catalytic specific nitrogen consumption rate (relative q*_N_), which could be explained by the variation of the ratio of specific carbohydrate production rate versus relative catalytic specific nitrogen consumption rate.The catalytic specific nitrogen consumption rate could be explained by this equation:

2 $$ \left(\frac{q{*}_N(t)}{q{*_N}_{\max }}\right) $$

**Figure 7:**
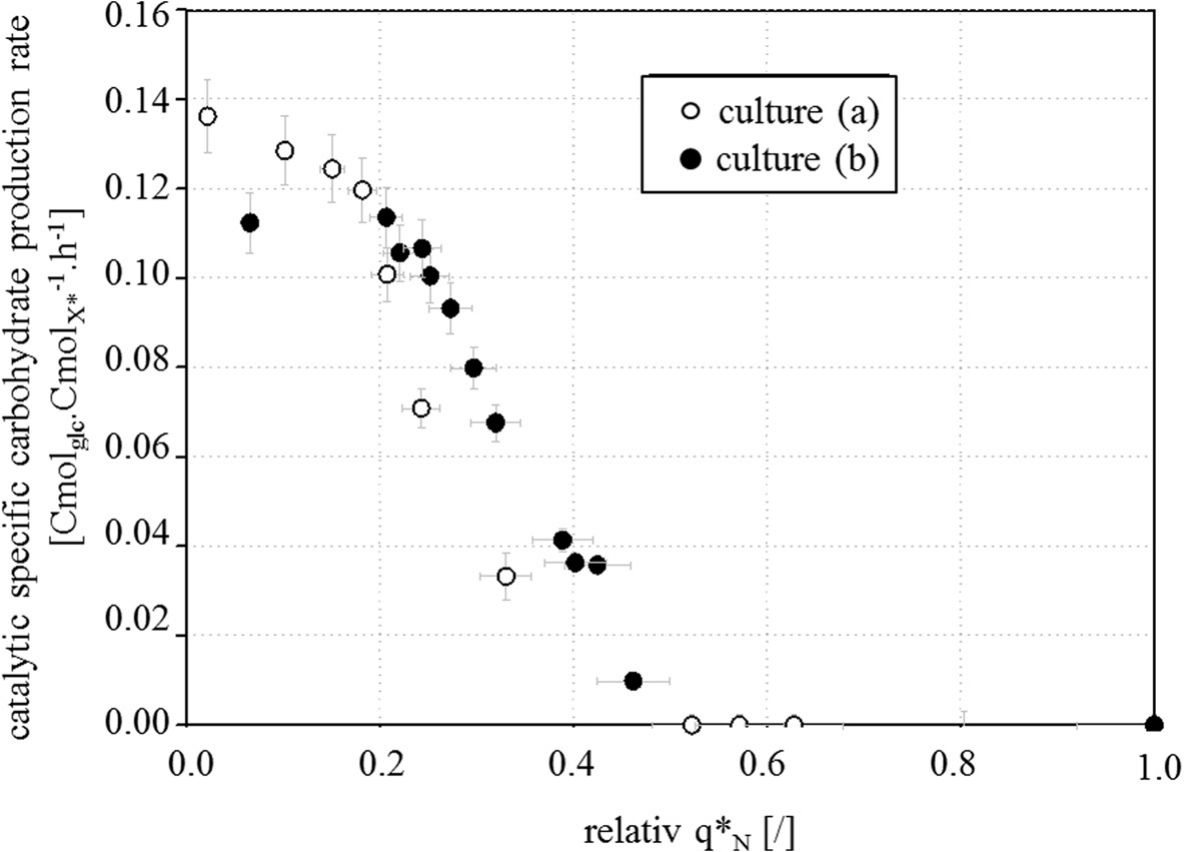
**Evolution of catalytic specific carbohydrate accumulation rate during fed-batch culture of** ***R. glutinis*** **at 30°C.** Evolution of q*_carbo_acc_ (Cmol_glc_acc_.Cmol_X*_^−1^.h^−1^)) versus relative catalytic specific nitrogen consumption rate (relative q*_N_; .[/]) with sudden nitrogen limitation (a; empty dot) and progressive limitation (b; solid dot).

This ratio was calculated during the first three hours of carbohydrate accumulation phase and is reported in Figure 7.

For both cultures, q^*^_carbo_acc_ vs relative q^*^_N_ evolution was sigmoidal. Maximum value of q*_carbo_acc_ was 0.14 for culture (a) and 0.11 Cmol_glc_acc_.Cmol_X*_^−1^.h^−1^ for culture (b).

Carbohydrate accumulation started as soon as q*_N_ was reduced by 50%, irrespective of the dynamics of nitrogen depletion; it occurred before the onset of lipid metabolism (at 18 hours for culture (a) and 22 hours for culture (b)).

In a very similar way, nitrogen starvation in *Saccharomyces cerevisiae* modified membrane lipid composition and intracellular polysaccharide accumulation [32-35]. Moreover, during culture of *S. cerevisiae* with nitrogen limitation and carbon excess, carbohydrates were continuously accumulated during batch and chemostat cultures [36,37]. Several works [33,36] demonstrated that *S. cerevisiae* accumulated trehalose and glycogen up to 25% (w/w) of dry cell mass during nitrogen limitation. In previous works, it was reported that *R. glutinis* could accumulate intracellular carbohydrate storage compounds, such as trehalose, glycogen, chitin and exopolysaccharides. Under carbon-limiting conditions, their mass content in trehalose varies from 0.5 to 3% (% glucose equivalent/dry cell weight) and from 0.3 to 0.1% in glycogen [38]. *R. glutinis* is also known to produce exopolysaccharides such as (1-3)-β- and (1-4)-β-D-mannopyranose [39]. Ghada *et al*. [40] reached a maximum polysaccharides concentration of 2.6 g.L^−1^, at pH 6.0 with an incubation period of 168 hours at 25°C and a biomass concentration of 8 g_X_.L^−1^. Moreover, Berthe *et al*. [18] observed that a pH decrease from 4.5 to 2 stimulated chitin synthesis (C_8_H_13_O_5_N)_n_ in the cell wall when *R. glutinis* was cultivated on glucose in batch mode; a pH increase back to 4.5 led to a decrease of the chitin content. Moulki and Bonaly [41] showed that the chitin content in the cell wall could be increased 21 fold, depending on the culture medium.

In these works, under carbon excess conditions, the intracellular trehalose and glycogen measurement (data not shown) showed that carbohydrate storage compounds were neither trehalose nor glycogen. No exocellular carbohydrates production was detected using the phenol-sulfuric acid method. Thus, we can assume that intracellular carbohydrates accumulated during fed-batch cultures could be due to chitin accumulation alone.

## Conclusion

Transitory carbohydrate accumulation in the oleaginous yeast *R. glutinis* was described and quantified when the nitrogen-to-carbon ratio in the feed decreased lower than 0.045 Nmol.Cmol^−1^. Intracellular carbohydrate content increased from 5 to 27% (Cmol_glc_.Cmol_X_^−1^) in 3 hours with a maximum catalytic specific production rate of 0.074 Cmol_glc_.Cmol_X*_^−1^.h^−1^. This storage occurred just before lipid accumulation metabolism started. Moreover, carbohydrates were accumulated regardless of the strategy applied for the nitrogen limitation set up (sudden and progressive). In these works, in carbon excess, neither trehalose or glycogen and exopolysaccharide were produced by *R. glutinis*. These observations led us to assume that intracellular carbohydrates accumulated during fed-batch cultures reported above, could be chitin alone. Lipid production performance in these works in fed-batch culture strategies with *R. glutinis* were higher than those reported in the literature until now; the catalytic specific lipid production rate was 0.07 Cmol_lip_.Cmol_X*_^−1^.h^−1^ with a volumetric lipid productivity of 1.05 g_lip_.L^−1^.h^−1^ and a substrate- (glucose)-to-lipid conversion yield of 0.42 Cmol_lip_.Cmol_S_^−1^. The maximum yeast concentration was 110 g_dcw_.L^−1^, containing 64% lipid (g_lip_.g_X_^−1^), and was reached in 50 hours. The best performance of microbial lipid production previously was reached with *Rhodosporidium toruloides* Y4, where a volumetric lipid productivity of 0.54 g_lip_.L^−1^.h^−1^ with a final yeast concentration of 106.5 g_dcw_.L^−1^ and a maximum lipid content of 67.5%_lip_ (g_lip_.g_X_^−1^) were reported by Li *et al*. [42].

The major accumulated fatty acids were oleic acid (47%), palmitic acid (29%), stearic acid (11%) and linoleic acid (9%). Compared to plant oil composition, microbial lipids from *R. glutinis* are of major interest for alternative oil production for biofuel applications.

## Abbreviations

DO: dissolved oxygen pressure compared to dissolved oxygen pressure with air saturation [% sat]
[i]: concentration of compound i [mol.L^−1^]
Μ: specific growth rate [h^−1^]
q_i_: specific production or consumption rate of compound i [Cmol_i_.Cmol_X_^−1^.h^−1^]
RQ: respiratory quotient $\left[\left(mo{l}_{C{O}_2}.\ \mathrm{reactor}\ {\mathrm{volume}}^{-1}.{\mathrm{h}}^{-1}\right).{\left(mo{l}_{O_2}.\ \mathrm{reactor}\ {\mathrm{volume}}^{-1}.{\mathrm{h}}^{-1}\right)}^{-1}\right]$
